# DFIQ, a Novel Quinoline Derivative, Shows Anticancer Potential by Inducing Apoptosis and Autophagy in NSCLC Cell and In Vivo Zebrafish Xenograft Models

**DOI:** 10.3390/cancers12051348

**Published:** 2020-05-25

**Authors:** Hurng-Wern Huang, Yung-Ding Bow, Chia-Yih Wang, Yen-Chun Chen, Pei-Rong Fu, Kuo-Feng Chang, Tso-Wen Wang, Chih-Hua Tseng, Yeh-Long Chen, Chien-Chih Chiu

**Affiliations:** 1 Institute of Biomedical Science, National Sun Yat-Sen University, Kaohsiung 804, Taiwan; sting@mail.nsysu.edu.tw; 2 Ph.D Program in Life Sciences, Kaohsiung Medical University; Kaohsiung 807, Taiwan; bowtobyd@gmail.com; 3 Department of Cell Biology and Anatomy, College of Medicine, National Cheng Kung University, Tainan 701, Taiwan; b89609046@gmail.com; 4 Department of Biotechnology, Kaohsiung Medical University, Kaohsiung 807, Taiwan; shiny_0224@yahoo.com.tw (Y.-C.C.); winnie840124@gmail.com (P.-R.F.); nickchang@welgene.com.tw (K.-F.C.); p796631@gmail.com (T.-W.W.); 5 School of Pharmacy, College of Pharmacy, Drug Development and Value Creation Research Center, Kaohsiung Medical University, Kaohsiung 807, Taiwan; chihhua@kmu.edu.tw; 6 Department of Medicinal and Applied Chemistry, Drug Development and Value Creation Research Center, Department of Medical Research, Kaohsiung Medical University Hospital, Kaohsiung Medical University, Kaohsiung 807, Taiwan; 7 Department of Biological Sciences, National Sun Yat-Sen University, Kaohsiung 804, Taiwan; 8 Center for Cancer Research, Kaohsiung Medical University, Kaohsiung 807, Taiwan; 9 The Graduate Institute of Medicine, Kaohsiung Medical University, Kaohsiung 807, Taiwan; 10 Department of Medical Research, Kaohsiung Medical University Hospital, Kaohsiung 807, Taiwan

**Keywords:** non-small-cell lung cancer (NSCLC), quinoline derivative, DFIQ, ROS, apoptosis, lysosome accumulation

## Abstract

Lung cancer is one of the deadliest cancers worldwide due to chemoresistance in patients with late-stage disease. Quinoline derivatives show biological activity against HIV, malaria, bacteriuria, and cancer. DFIQ is a novel synthetic quinoline derivative that induces cell death in both in vitro and in vivo zebrafish xenograft models. DFIQ induced cell death, including apoptosis, and the IC_50_ values were 4.16 and 2.31 μM at 24 and 48 h, respectively. DFIQ was also found to induce apoptotic protein cleavage and DNA damage, reduce cell cycle-associated protein expression, and disrupt reactive oxygen species (ROS) reduction, thus resulting in the accumulation of superoxide radicals. Autophagy is also a necessary process associated with chemotherapy-induced cell death. Lysosome accumulation and lysosome-associated membrane protein-2 (LAMP2) depletion were observed after DFIQ treatment, and cell death induction was restored upon treatment with the autophagy inhibitor 3-methyladenine (3-MA). Nevertheless, ROS production was found to be involved in DFIQ-induced autophagy activation and LAMP2 depletion. Our data provide the first evidence for developing DFIQ for clinical usage and show the regulatory mechanism by which DFIQ affects ROS, autophagy, and apoptosis.

## 1. Introduction

Lung cancer is one of the deadliest cancers worldwide [1], and approximately 85% of all lung cancer cases are non-small-cell lung carcinoma (NSCLC), including adenocarcinoma, squamous cell carcinoma, and large cell carcinoma [2]. The low survival rate of patients with late-stage NSCLC and chemoresistance result in poor patient prognosis [3]. Many cancer therapies target lung cancer, and the most common treatments are surgery, targeted therapy, and chemotherapy [4].

**Figure 1:**
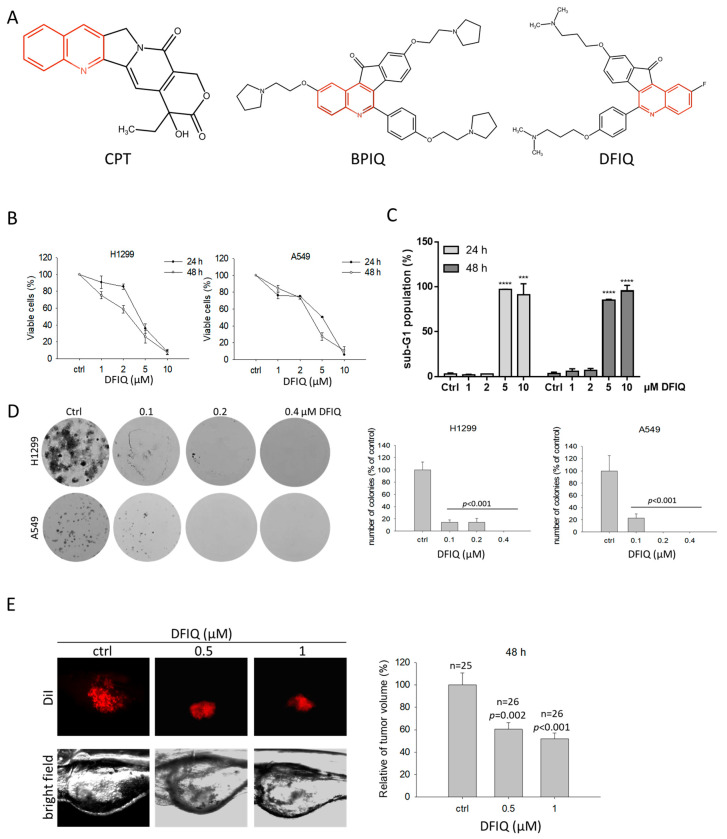
DFIQ inhibited non-small-cell lung carcinoma (NSCLC) cell growth in vitro and in vivo. (**A**) Chemical structures of camptothecin (CPT), BPIQ, and DFIQ. The quinoline scaffold is marked in red. (**B**) The NSCLC cell lines A549 and H1299 were treated with different doses of DFIQ, and cell viability was measured at 24 and 48 h after treatment. (**C**) We measured the proportion of sub-G1 cells among DFIQ-treated H1299 cells at 24 and 48 h. *** *p* < 0.001, **** *p* < 0.0001 compared to the control group. (**D**) Measurement and quantification of colony formation of A549 and H1299 cells treated with DFIQ. (**E**) Growth inhibitory activity of DFIQ in NSCLC cells in the zebrafish xenograft model at 48 h after treatment.

Compounds with a quinoline scaffold show biological activity against HIV [5,6], malaria [7,8], Alzheimer’s disease [9], and cancer [10,11]. Camptothecin (CPT) is a well-known quinoline derivative found in the bark and stems of *Camptotheca acuminate* [12] and shows anticancer potential by inducing DNA double-strand breaks and apoptosis [13]. Decades of research has resulted in the development of several CPT derivatives, such as irinotecan [14] (CPT-11) and belotecan [15] (CKD-602), that have been utilized in clinical cancer therapy. We have developed two quinoline derivatives: 2,9-bis[2-(pyrrolidin-1-yl)ethoxy]-6-{4-[2-(pyrrolidin-1-yl)ethoxy]phenyl}-11*H*-indeno[1,2-*c*]quinolin-11-one (BPIQ) and 9-[3(dimethylamino)propoxy]-6-{4-[3-(dimethylamino)propoxy]phenyl}-2-fluoro-11*H*-indeno[1,2-*c*]quinolin-11-one (DFIQ) [16,17] (Figure 1A). BPIQ was found to induce reactive oxygen species (ROS) production, Endoplasmic Reticulum (ER) stress, and apoptosis and to exert high anticancer potential in lung cancer, retinoblastoma, and hepatocellular carcinoma by inducing ER stress and apoptosis, and inhibiting migration [18,19,20,21]. DFIQ is a novel synthetic quinoline derivative that shares a similar structure with BPIQ. Based on the anticancer function of BPIQ, DFIQ has strong potential against NSCLC and could induce cell death. Thus, in this study, we investigated the anticancer ability of DFIQ by monitoring cell growth, migration, and apoptosis. Furthermore, we clarified the possible anticancer mechanism of DFIQ in NSCLC.

## 2. Results

### 2.1. DFIQ Shows Anti-NSCLC Potential

To determine the anticancer potential of DFIQ in NSCLC, we treated H1299 and A549 NSCLC cell lines with different DFIQ concentrations and measured cell viability. Significant cell death was observed in the groups treated with over 5 μM DFIQ (Figure 1B). As shown in Table 1, the IC_50_ values of DFIQ in H1299 and A549 cells were 4.16 and 5.06 μM after 24 h of treatment and 2.81 and 3.53 μM after 48 h of treatment, respectively. To determine the type of DFIQ-induced cell damage, the percentage of sub-G1 cells was measured after DFIQ treatment. A rapid increase in the sub-G1 population was observed in H1299 cells treated with over 5 μM DFIQ (Figure 1C, Figure S1A). Additionally, colony formation assays were performed using DFIQ-treated H1299 and A549 NSCLC cells to reveal the ability of a single cell to grow into a colony. Cells exposed to a relatively low concentration of DFIQ lost the ability to grow from a single cell into a colony (Figure 1D). In addition, DFIQ inhibited cell migration at concentrations lower than 5 μM (Figure S1B,C). A zebrafish xenograft model was utilized to examine the growth inhibitory effect of DFIQ in vivo. H1299 cells were implanted into the yolk of zebrafish larvae for 72 h, followed by incubation with 0, 0.5, or 1 μM DFIQ for 48 h. Consistently, the tumor volumes were significantly decreased after DFIQ treatment (Figure 1E). The results indicated that DFIQ has strong potential as an anticancer therapy.

### 2.2. Apoptosis is Associated with DFIQ-Induced Cell Death

**Figure 2:**
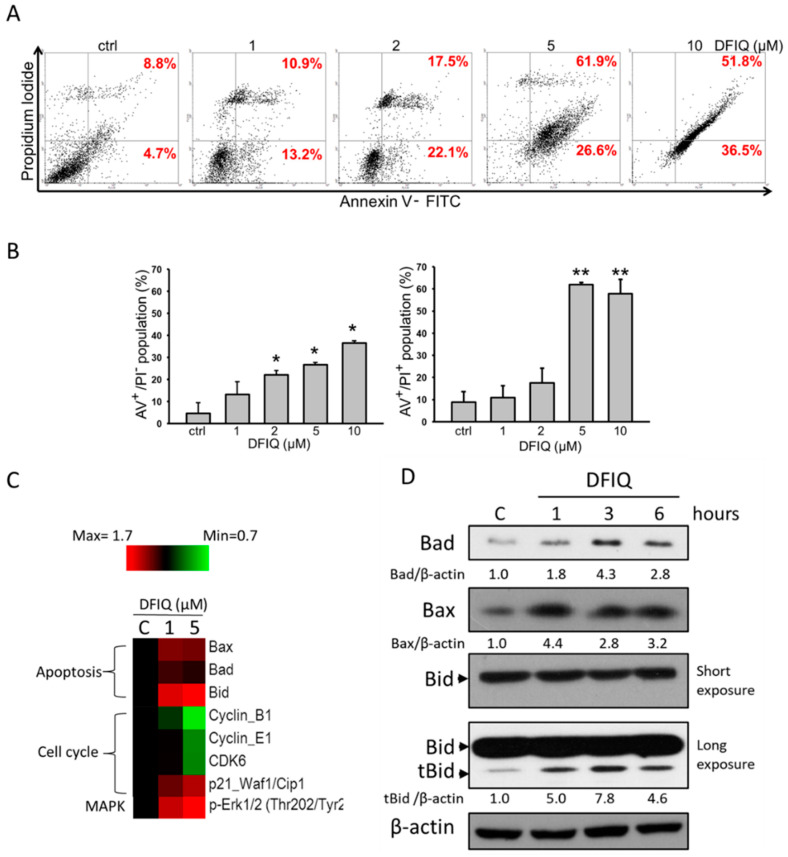
DFIQ treatment induced apoptosis of lung cancer cells. (**A**) H1299 cells were treated with control or 1, 2, 5, or 10 μM DFIQ for 24 h and subjected to Annexin V/PI staining to analyze the apoptotic cells. (**B**) Quantification of early apoptotic cells (fourth quadrant) and late apoptotic cells (first quadrant) in (**A**). (**C**) Micro-Western array analysis of the expression changes in apoptotic and cell cycle proteins after DFIQ treatment in H1299 cells. (**D**) Western blot analysis demonstrating the expression of the apoptotic proteins Bad, Bax, and tBid after DFIQ treatment. β-Actin was measured as a loading control. ** *p* < 0.01, * *p* < 0.05 compared with the control group. The uncropped blots and molecular weight markers of Figure 2D are shown in Figure S6.

The results showed that DFIQ increased the sub-G1 population of NSCLC cells, suggesting that DFIQ-mediated cell death was associated with apoptosis (Figure 1C) [22]. To determine the mechanism of DFIQ-induced cell death, Annexin V/PI double staining was performed, which indicated apoptotic cell death after DFIQ treatment. A significant increase in the number of Annexin V^+^ cells was found in DFIQ-treated cells, especially in those treated with over 5 μM DFIQ (Figure 2A). DFIQ treatment initiated apoptosis at relatively low concentrations and induced early apoptosis. In addition, a rapid increase in cell death was observed, which was caused by late apoptosis in cells treated with over 5 μM DFIQ (Figure 2B). Due to the function of CPT, we also quantified DFIQ-induced DNA damage with γH2AX [23]. Thirty percent of cells treated with DFIQ were found to have damaged DNA (Figure S2). As DFIQ-induced cell death was associated with apoptosis, we screened changes in the expression of apoptotic and cell cycle-associated proteins with a Micro-Western blot array. Bax, Bad, and Bid are apoptotic proteins that induce apoptosis when cells are subject to the extrinsic pathway, which includes proteins such as Fas, TNF, and TRAIL [24,25]. According to the Micro-Western blot array results, DFIQ treatment induced Bax, Bid, and Bad expression and initiated apoptosis (Figure 2C). On the other hand, the cell cycle-associated proteins cyclin B1, cyclin E1, and CDK6, which mediate mitosis, S phase, and G1-S phase transition [26], respectively, were downregulated after DFIQ treatment (Figure 2C). Additionally, p21, which inhibits the function of the cyclin E1/Cdk2 complex and results in cell cycle arrest [27], was upregulated during DFIQ treatment (Figure 2C). We also examined the expression of the proapoptotic proteins Bad, Bax, and the truncated form of Bid (tBid) in DFIQ-treated H1299 cells. The significant upregulation in expression during DFIQ treatment (Figure 2D) revealed that DFIQ-induced cell death is associated with cell cycle-associated protein dysfunction and apoptotic protein induction and thus initiates apoptosis.

### 2.3. DFIQ Disrupted the Metabolic ROS Clearance Axis and Induced Cell Apoptosis

**Figure 3:**
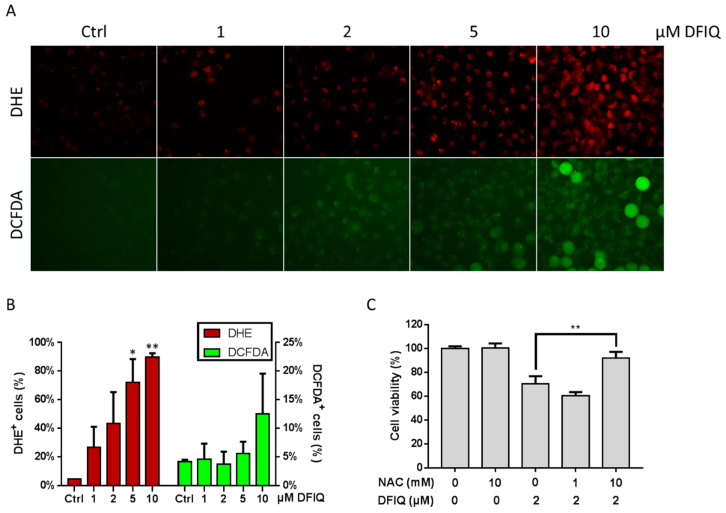
Reactive oxygen species (ROS) formation is associated with DFIQ-induced apoptosis. (**A**) H1299 cells treated with different concentrations of DFIQ for 6 h were stained with dihydroethidium (DHE) or 2’,7’-dichlorofluorescein diacetate (DCFDA) to detect O_2_^−^ or H_2_O_2_ formation, respectively. (**B**) Quantification of O_2_^−^ or H_2_O_2_ formation in (**A**). (**C**) The viability of cells pretreated with the ROS inhibitor N-acetylcysteine (NAC) prior to treatment with DFIQ was monitored. ** *p* < 0.01, * *p* < 0.05 compared with the control group.

ROS are usually small molecules with high reactivity and short half-lives and include oxygen-derived free radicals, hydroxyls, and nonradical molecules, such as superoxide anions (O_2_^−^), hydroxyl radicals (OH), and hydrogen peroxide (H_2_O_2_) [28]. ROS are also common factors that regulate apoptosis and cause organelle damage [29,30]. Thus, ROS are potential DFIQ targets to induce apoptosis. In our study, we used dihydroethidium (DHE) and 2’,7’-dichlorofluorescein diacetate (DCFDA) to measure the levels of O_2_^−^ and H_2_O_2_, respectively. Considerable superoxide anion levels were found in over 60% of cells after 5 μM DFIQ treatment and in over 80% of cells after 10 μM DFIQ treatment (Figure 3A,B). Interestingly, the levels of H_2_O_2_, a low toxicity transition molecule within O_2_^−^ metabolism that is catalyzed by the superoxide dismutase (SOD) family, were not different between the control and DFIQ treatment groups (Figure 3A,B). Nevertheless, we found no significant difference in the expression of the SOD family of proteins between the control and DFIQ treatments (Figure S3). To determine whether DFIQ-induced cell death was associated with ROS production, we treated cells with the ROS inhibitor N-acetylcysteine (NAC), which is considered an antioxidant [31], and measured cell survival after DFIQ treatment. The results showed that NAC ameliorated cell death caused by DFIQ (Figure 3C). The results suggested that ROS play a role in DFIQ-induced apoptosis and that DFIQ treatment might be associated with dysfunction of the process of removing ROS.

### 2.4. DFIQ-Induced Apoptosis is Initiated by Autophagy

**Figure 4:**
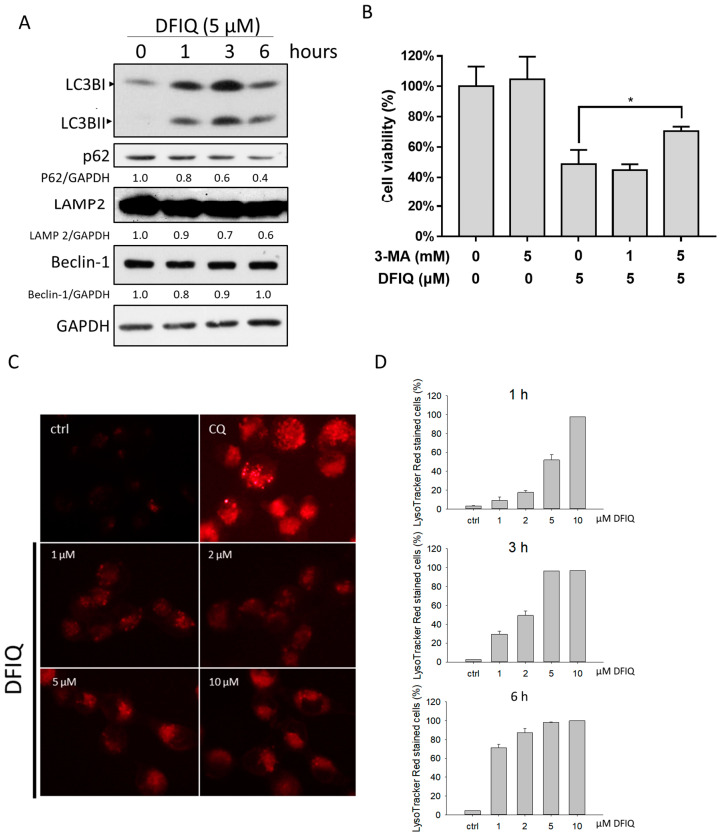
Autophagy might be involved in DFIQ-mediated apoptosis. (**A**) Western blot analysis of autophagy-associated proteins after the indicated DFIQ treatment. (**B**) H1299 cells were pretreated with the autophagy inhibitor 3-methyladenine (3-MA) before treatment with DFIQ, and viability was monitored. (**C**) Lysosomes were stained with LysoTracker Red to observe lysosome formation after DFIQ treatment for 6 h. Chloroquine (CQ) served as a positive control. (**D**) Quantification of lysosome formation at different time points of DFIQ treatment. * *p* < 0.05 compared with the control group. The uncropped blots and molecular weight markers of Figure 4A are shown in Figure S7.

Autophagy is a standard cellular procedure that eliminates damaged organelles and misfolded proteins to adapt to environmental signals, such as nutrient depletion and hypoxia [32]. ROS are considered an early inducer of autophagy and can promote autophagic cell death [32]. In turn, ROS-induced autophagy contributes to the removal of ROS in response to stressors [33]. Therefore, we measured the expression of the autophagic protein LC3II, which is cleaved from the LC3I protein during autophagosome formation. LC3II upregulation was observed in cells treated with DFIQ at all time points tested (Figure 4A). In addition, the levels of p62, which is degraded during autophagosome formation [34], were downregulated (Figure 4A). The results showed that autophagy was activated after DFIQ treatment. On the other hand, we measured cell viability after treatment with DFIQ and the autophagy inhibitor 3-MA [35] to evaluate whether autophagy is involved in DFIQ-induced cell death and found that DFIQ-induced cell death was restored after 3-MA treatment (Figure 4B). In addition, the expression of LAMP2, a protein involved in autophagosome and lysosome fusion [36], was downregulated after DFIQ treatment (Figure 4A). Chloroquine (CQ) is a chemical utilized to block autophagosome-lysosome fusion and causes the accumulation of lysosomes [37]. Due to DFIQ-induced LAMP2 depletion, rapid lysosome formation was observed after DFIQ treatment, indicating that DFIQ disrupted autophagosome-lysosome fusion (Figure 4C,D). The localization of LC3 and LAMP2 indicates the fusion of autophagosomes and lysosomes, and colocalization of LC3-GFP and LAMP2-DsRed was used to monitor autophagolysosome formation, which was observed under control conditions but was disrupted after DFIQ treatment (Figure S4).

### 2.5. Crosstalk between DFIQ-Induced Autophagy and ROS Production

**Figure 5:**
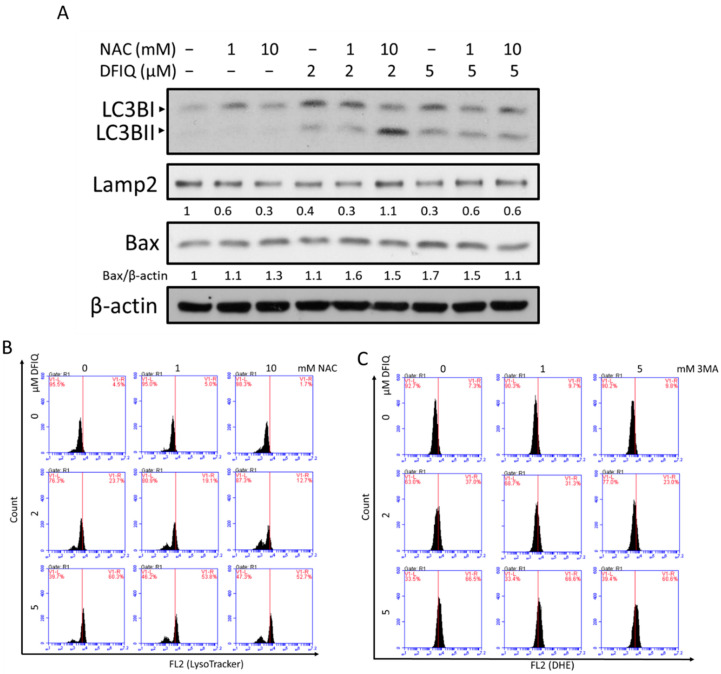
Crosstalk between autophagy and ROS during DFIQ treatment. (**A**) Western blot analysis of the expression of autophagic and apoptotic proteins after DFIQ treatment for 6 h. (**B**) Lysosome accumulation after cotreatment with DFIQ and NAC for 6 h. (**C**) Superoxide formation after cotreatment with DFIQ and the autophagy inhibitor 3-MA for 6 h. The uncropped blots and molecular weight markers of Figure 5A are shown in Figure S8.

DFIQ inhibited cell growth via autophagy and ROS production and upregulated the expression of apoptotic proteins in NSCLC cells. The association between ROS production and autophagy is also important in elucidating the mechanism of DFIQ-induced apoptosis. Thus, cells were treated with DFIQ and NAC to identify alterations in the expression levels of autophagic proteins. NAC treatment reversed the DFIQ-induced upregulation of LC3II expression and downregulation of p62 expression (Figure 5A and Figure S5), indicating that increased ROS levels during DFIQ treatment enhanced autophagy activity. In addition, Lamp2 expression was rescued from DFIQ-induced downregulation upon NAC treatment, restoring autophagosome-lysosome fusion (Figure 5A). The expression of the apoptotic proteins Bad and Bax was also downregulated after blocking ROS accumulation (Figure 5A and Figure S5). NAC treatment also mitigated lysosome accumulation during DFIQ treatment (Figure 5B), and 3-MA reduced DFIQ-mediated ROS production (Figure 5C). The results indicated that DFIQ-mediated ROS production resulted in the loss of Lamp2 expression and caused the accumulation of lysosomes and that inhibiting autophagy reduced the formation of ROS.

## 3. Discussion

Chemotherapy plays a critical role in NSCLC. Quinoline derivatives have been used for anticancer therapy since ME Wall et al. isolated CPT from *Camptotheca acuminate* [12]. Many quinoline derivatives, such as CPT [12], topotecan [38], irinotecan [14], and belotecan [15], were developed, have shown anticancer potential [39] by driving cancer apoptosis, and have been utilized in clinical therapy. According to previous studies, irinotecan showed mild cytotoxicity to NSCLC, with an IC_50_ value from 16 to 218 μM depending on the exposure duration and pH value [40]. Meanwhile, topotecan showed potent anticancer ability with a low IC_50_ value in lung adenocarcinoma (1 μM for 72 h) and lung squamous cell carcinoma (0.1 μM for 72 h) [41]. Although topotecan showed great cytotoxicity to NSCLC, desensitization of cells to topotecan was also observed [42]. The novel quinoline derivatives BPIQ and DFIQ showed cytotoxicity against NSCLC, and the IC_50_ values of BPIQ were 1.96 μM (24 h) and 1.3 μM (48 h) [18]. Although BPIQ and topotecan showed a better IC_50_ value for NSCLC, DFIQ was less cytotoxic at high concentrations and arrested a considerable percentage cells in sub-G1 phase within 6 h. Moreover, DFIQ inhibited cell migration ability at low concentrations that were less cytotoxic toward NSCLC. Therefore, DFIQ can be considered to be a bifunctional anticancer compound that both induces cell death and inhibits cell migration.

Due to their size, high genetic similarity, embryonic transparency, low cost, and rapid development, zebrafish have been widely utilized in cancer-associated research in recent years [43]. The zebrafish xenograft assay has been used to investigate tumor viability, invasion, and angiogenesis [43]. In addition, zebrafish can also serve as a model to evaluate the interactions between the environment and cancer cells and the side effects of compounds. Although DFIQ showed an inhibitory effect on lung cancer cells in the zebrafish model, zebrafish viability was rapidly decreased with a high dose of DFIQ (over 5 μM). This result indicates that DFIQ should be used responsibly during cancer chemotherapy.

DFIQ showed high potential against NSCLC by inducing ROS and autophagy to initiate apoptosis. In addition, superoxide accumulation was observed in cells treated with DFIQ. Superoxide accumulation usually occurs when SOD protein activity is dysfunctional and is associated with growth arrest and apoptosis [44]. Although the expression of CuSOD (SOD1) and MnSOD (SOD2) showed no significant difference during DFIQ treatment (Figure S3), the ROS reduction process was still blocked (Figure 3A,B). The function of SOD is usually associated with metal ions, such as Fe^2+^, Cu^2+^, Mn^2+^, and Zn^2+^, which play a vital role during ROS reduction [45]. Interference with ion metabolism and inhibition of SOD function are possible targets for overcoming DFIQ-induced blockage of ROS reduction.

Autophagy is a vital procedure associated with cell survival as well as programmed cell death [46]. During this process, autophagosomes fuse with lysosomes to form autophagolysosomes and degrade their contents [47]. Lysosome accumulation is a common characteristic of disrupted the fusion of autophagosome and lysosome. Inhibition of this process was found to induce apoptosis, which disrupts the cell survival mechanism activated by autophagy [48,49,50]. Therefore, DFIQ-induced apoptosis might be associated with lysosome accumulation and could be restored by autophagy inhibition, which decreases lysosome formation (Figure 4B,C).

In addition, the crosstalk between autophagy and ROS formation during apoptosis initiation is vital to elucidate the mechanism of DFIQ-induced apoptosis. LAMP2 expression was observed to be reduced during ischemia-reperfusion injury, which results in the considerable production of ROS [51,52] and induction of lysosome and autophagosome accumulation [53].

Autophagy plays a vital role in both the proapoptotic pathway and prosurvival pathway depending on the injury. The data showed that the inhibition of ROS production reduced cell death (Figure 3C) but induced autophagy, which resulted in increased LC3BII expression and reduced p62 expression (Figure 5A,B). Some factors that regulate autophagy function are responsible for switching autophagy from the proapoptotic to the prosurvival pathway. According to research published by X. Ou and H.E. Broxmeyer, knocking out Sirtuin-1 (SIRT1), an NAD-dependent deacetylase, inhibited the prosurvival function of autophagy during oxidative stress [54]. In addition, SIRT1 expression is associated with oxidative stress and is involved in apoptosis [55,56]. Therefore, we suggest that DFIQ-induced ROS formation inhibits SIRT1 expression and that autophagy exerts a proapoptotic function; however, upon ROS inhibition, SIRT1 is re-expressed and switches from a proapoptotic function to a prosurvival function. Thus, SIRT1 might play a vital role in DFIQ-induced apoptosis.

## 4. Materials and Methods

### 4.1. Cell Culture

The human NSCLC cell lines H1299, A549 and H460 were obtained from American Type Culture Collection (ATCC; Manassas, VA, USA) and maintained in DMEM/F12 (3:2) supplemented with 10% FBS, 2 mM glutamine, and antibiotics at 37 °C in air with 5% CO_2_.

### 4.2. Cell Viability

Cell viability was determined by the trypan blue exclusion assay. Briefly, cells were seeded and treated with DFIQ, NAC, or 3-MA for a determined time and exposed to 0.2% trypan blue dye to count the number of live cells.

### 4.3. Colony Formation Assay

A total of 1000 H1299 or A549 cells were seeded into a 6-well plate, incubated for 24 h, and treated with DFIQ for 11 days. After incubation, the colonies were fixed with 4% paraformaldehyde (PFA) and stained with 0.1% Giemsa stain overnight.

### 4.4. Apoptosis Determination

To monitor the apoptosis-inducing potential of DFIQ, Annexin V/PI double staining was used to detect the inner membrane leaflet marker phosphatidylserine (PS), which translocates to the outer leaflet during apoptosis, and DNA, which is stained during only late apoptosis or necrosis. Briefly, cells were seeded onto dishes and treated with 0, 1, 2, 5, or 10 μM DFIQ for 24 h. After treatment, the cells were harvested, stained with an Annexin V staining kit according to a standard procedure, and analyzed with an LSR II flow cytometer (BD Biosciences, San Jose, CA, USA) and FlowJo 7.6.1 software (TreeStar, Inc., Ashland, OR, USA).

### 4.5. Micro-Western Blot Array

A high-throughput Micro-Western array was performed at the Micro-Western Array core facility of the National Health Research Institutes (NHRI) of Taiwan, as previously described [57]. Briefly, lysates collected from cells treated with DFIQ for 6 h were washed with PBS, resuspended in lysis buffer, and sent to the NHRI for high-throughput screening of 48 target proteins.

### 4.6. Western Blot Analysis

Cells were lysed with lysis buffer and centrifuged at 4 °C to collect the protein contained in the supernatant. The total protein mass was determined by the bicinchoninic acid (BCA) protein assay kit (Pierce, Rockford, IL, USA). Equal amounts of total protein lysate were separated by SDS-polyacrylamide gel electrophoresis (SDS-PAGE) and electrotransferred to polyvinylidene difluoride (PVDF, PALL, Ann Arbor, USA) membranes. The PVDF membranes were blocked with 5% nonfat milk in TBS-T buffer (TBS buffer containing 0.1% Tween 20) for 1 h and hybridized with primary antibodies followed by HRP-conjugated secondary antibodies. HRP activity was detected with an enhanced chemiluminescence (ECL) detection kit (Amersham Piscataway, NJ, USA).

### 4.7. ROS Detection

Dihydroethidium (DHE) and 2’,7’-dichlorofluorescein diacetate (DCFDA) were used to determine the formation of the intracellular ROS superoxide (O_2_^−^) and hydrogen dioxide (H_2_O_2_), respectively. Treated cells were incubated with DHE or DCFDA, and then the fluorescence intensity was measured. The data were analyzed by FlowJo 7.6.1 (TreeStar, Inc) and SigmaPlot 11.0 software (Systat Software, San Jose, CA, USA).

### 4.8. LysoTracker Red Assay

Lysosomes were detected with LysoTracker Red dye (Invitrogen, Carlsbad, CA, USA) according to the manufacturer’s instructions. Briefly, the treated cells were exposed to 1 μM LysoTracker Red for 30 min and then analyzed on an LSR II flow cytometer (BD Biosciences).

### 4.9. Statistical Analysis

Differences between groups were analyzed in at least triplicate experiments, and the results were analyzed by one-way analysis of variance (ANOVA). *p* < 0.05 was considered significant.

### 4.10. Zebrafish Xenograft Assay

The zebrafish protocol was performed as described previously [58]. Zebrafish (*Danio rerio*) were kept at 28 °C in aquaria with a 14-/10-h light/dark cycle. Zebrafish larvae were kept in an incubator at a constant temperature. The zebrafish assay complied with the principles of 3Rs (Reduction, Replacement and Refinement) and the Approval Code. KMU-IACUC-104109 by Institutional Animal Care and Use Committee (IACUC) of Kaohsiung Medical University Hospital, Kaohsiung, Taiwan.

## 5. Conclusions

**Figure 6:**
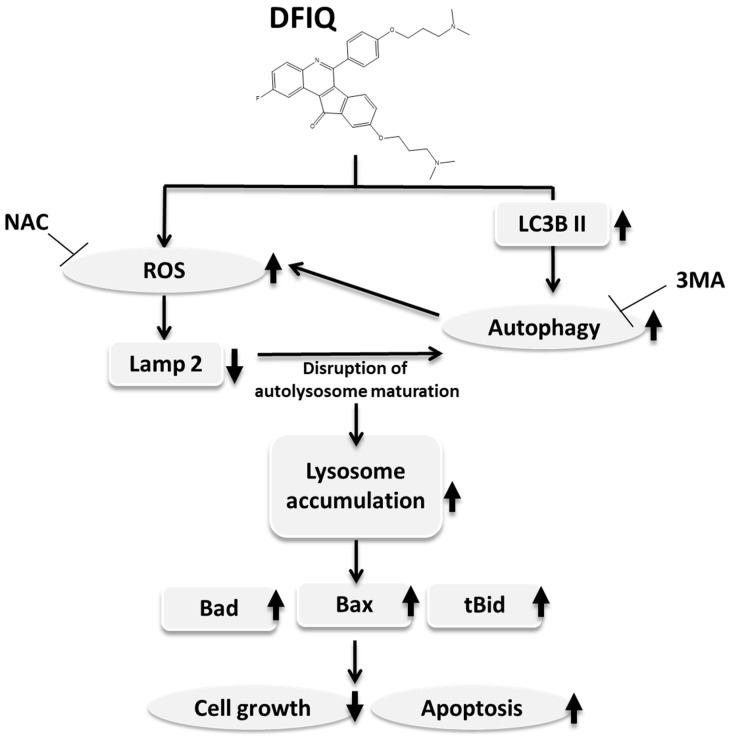
Proposed mechanism of DFIQ-induced apoptosis in lung cancer cells. DFIQ exerts anticancer potential that regulates the apoptosis of lung cancer cells. During DFIQ treatment, the level of O_2_^−^ was significantly increased, but the metabolic function of O_2_^−^ was disrupted, and the expression of the autophagolysosome formation-associated protein LAMP2 was downregulated, resulting in disruption of autophagolysosome maturation. On the other hand, DFIQ also induced the process of autophagy associated with ROS formation. Due to the disruption in autolysosome maturation and the induction of autophagy, lysosomes accumulate in cells treated with DFIQ, eventually inducing the expression of the apoptotic proteins Bad, Bax, and tBid and thus causing apoptosis.

This study suggests that DFIQ exerts anticancer potential in vivo and in vitro and can induce apoptosis. DFIQ-induced apoptosis is associated with lysosome accumulation and the induction of the expression of apoptosis factors, such as Bax, Bad, and tBid. In addition, ROS production plays a vital role in DFIQ-induced cell death by causing Lamp2 downregulation, which results in lysosome accumulation, and is regulated by autophagy (Figure 6). This research reveals the partial mechanism by which DFIQ induces apoptosis and proposes a basis for pursuing DFIQ-mediated chemotherapy in NSCLC.

## Figures and Tables

**Table 1:** The IC_50_ values for DFIQ in H1299 and A549 cells.

Cell Line	IC_50_ (μM)
H1299	24 h: 4.16 μM
	48 h: 2.81 μM
A549	24 h: 5.06 μM
	48 h: 3.53 μM

## Supplementary Materials

The following are available online at https://www.mdpi.com/2072-6694/12/5/1348/s1, Figure S1: DFIQ inhibited cell growth and cell migration. (A) The cell cycle distribution of cells treated with 5 μM DFIQ was monitored at 1, 3, 6, 12, and 24 h with flow cytometry. (B) Cell migration was measured with the wound healing assay. Cells were treated with 0.5, 1, 2, or 5 μM DFIQ for 6 h, and gap closure was measured at 16 h after scratching. (C) Quantification of the area of the wound in (B). ** *p* < 0.05, ** *p* < 0.01, compared to the control group, Figure S2: DFIQ induced DNA damage. (A) DNA damage was visualized with γH2AX staining in cells treated with 2 and 5 μM DFIQ. (B) Quantification of γH2AX-positive cells in (A), Figure S3: No alterations in the expression of the SOD family of proteins was observed after DFIQ treatment. Western blot analysis of SOD family protein expression after DFIQ treatment, Figure S4: DFIQ inhibited autophagosome-lysosome fusion. We labeled the autosomal protein LC3 with GFP (green) and the lysosomal protein LAMP2 with DsRed (red) and observed colocalization of LC3 and LAMP2 after 2 μM DFIQ treatment, Figure S5: Crosstalk between autophagy and ROS during DFIQ treatment. Western blot analysis of p62 and Bad expression after 5 μM DFIQ and NAC treatment.
