# Supplementary material for: DFIQ, a Novel Quinoline Derivative, Shows Anticancer Potential by Inducing Apoptosis and Autophagy in NSCLC Cell and In Vivo Zebrafish Xenograft Models
Source: Cancers (Basel). 2020 May 25;12(5):1348. doi: 10.3390/cancers12051348 (PMC7281296; doi:10.3390/cancers12051348)
Supplement: Supplementary file 1 [file cancers-12-01348-s001.pdf]

Supplementary Materials

DFIQ, a Novel Quinoline Derivative, Shows Anticancer Potential by Inducing Apoptosis and Autophagy in NSCLC Cell and In Vivo Zebrafish Xenograft Models

Hurng-Wern Huang, Yung-Ding Bow, Chia-Yih Wang, Yen-Chun Chen, Pei-Rong Fu, Kuo-Feng Chang, Tso-Wen Wang, Chih-Hua Tseng, Yeh-Long Chen and Chien-Chih Chiu

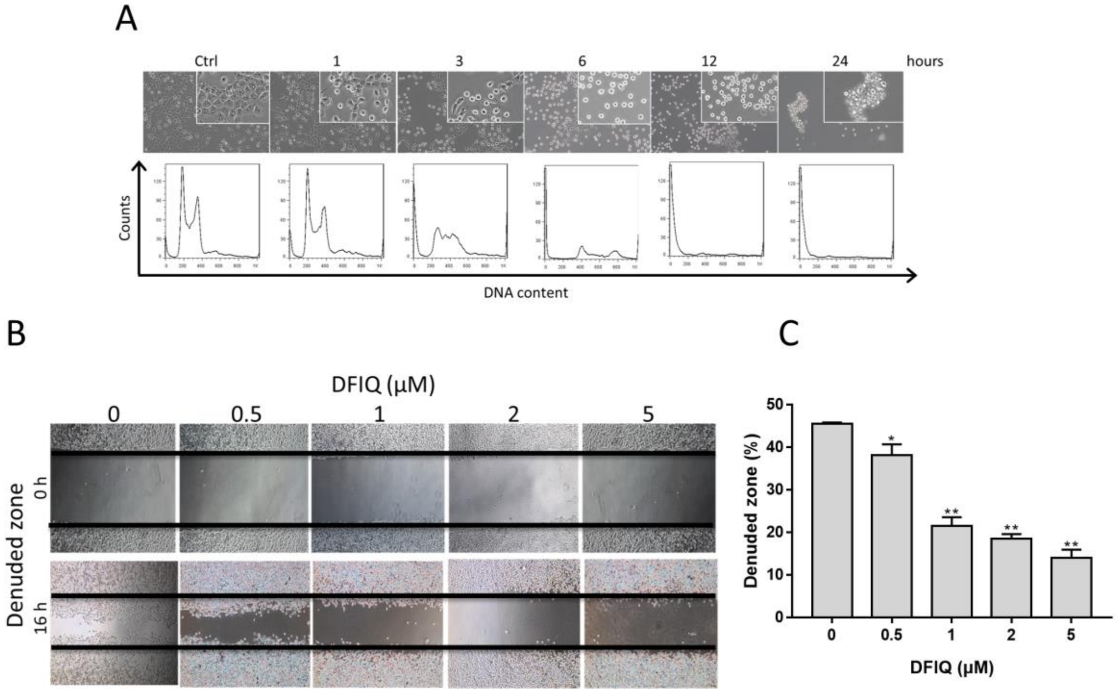

Figure S1. DFIQ inhibited cell growth and cell migration. (**A**) The cell cycle distribution of cells treated with 5 µM DFIQ was monitored at 1, 3, 6, 12, and 24 h with flow cytometry. (**B**) Cell migration was measured with the wound healing assay. Cells were treated with 0.5, 1, 2, or 5 µM DFIQ for 6 h, and gap closure was measured at 16 h after scratching. (**C**) Quantification of the area of the wound in (B). * $p < 0.05$, ** $p < 0.01$, compared to the control group.

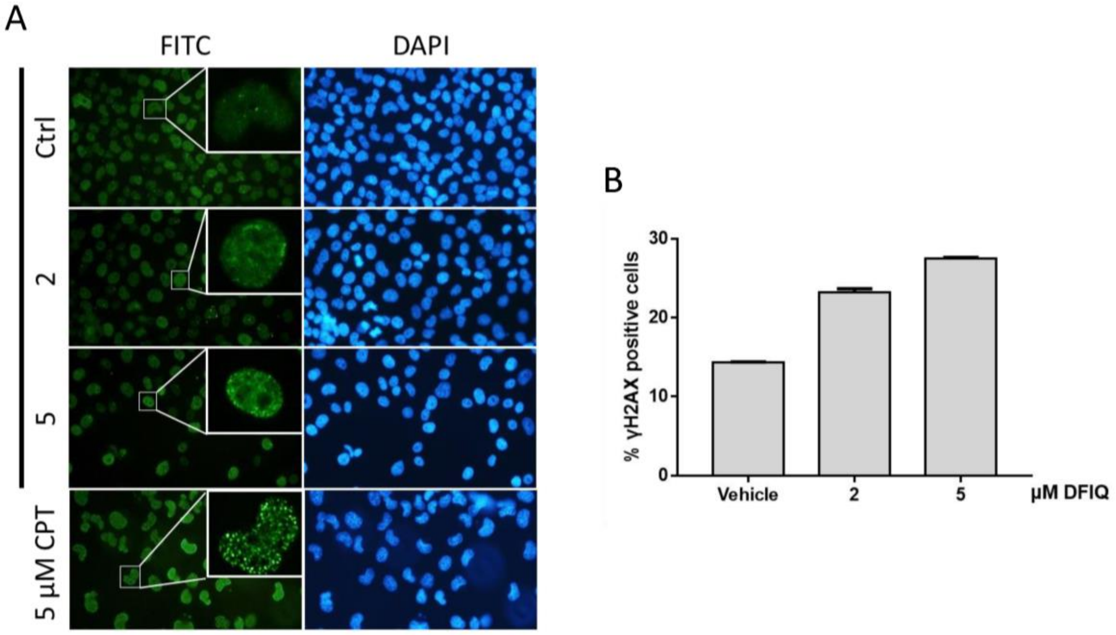

Figure S2. DFIQ induced DNA damage. (**A**) DNA damage was visualized with γH2AX staining in cells treated with 2 and 5 µM DFIQ. (**B**) Quantification of γH2AX-positive cells in (A).

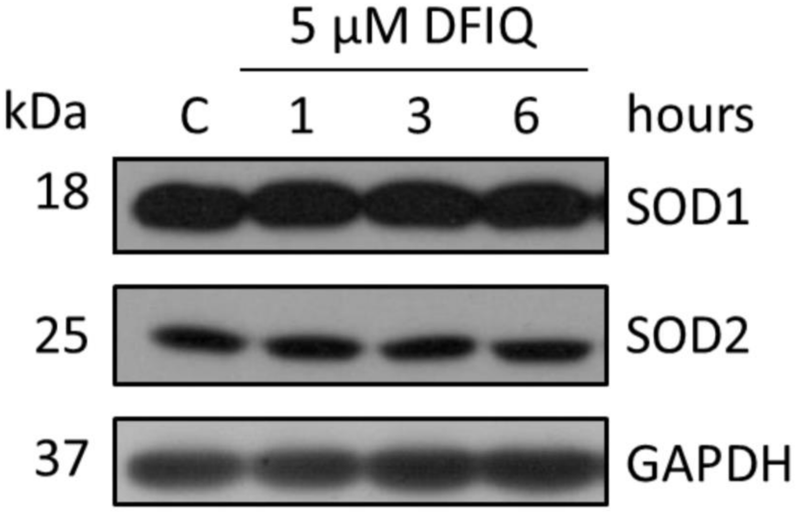

Figure S3. No alterations in the expression of the SOD family of proteins was observed after DFIQ treatment. Western blot analysis of SOD family protein expression after DFIQ treatment.

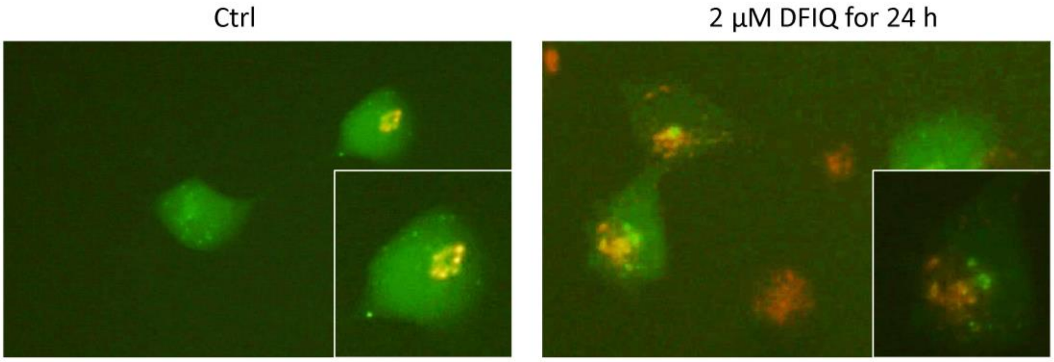

Figure S4. DFIQ inhibited autophagosome-lysosome fusion. We labeled the autosomal protein LC3 with GFP (green) and the lysosomal protein LAMP2 with DsRed (red) and observed colocalization of LC3 and LAMP2 after 2 μM DFIQ treatment.

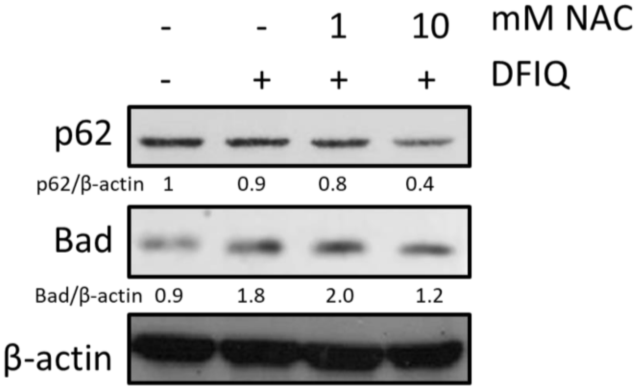

Figure S5. Crosstalk between autophagy and ROS during DFIQ treatment. Western blot analysis of p62 and Bad expression after 5 μM DFIQ and NAC treatment.

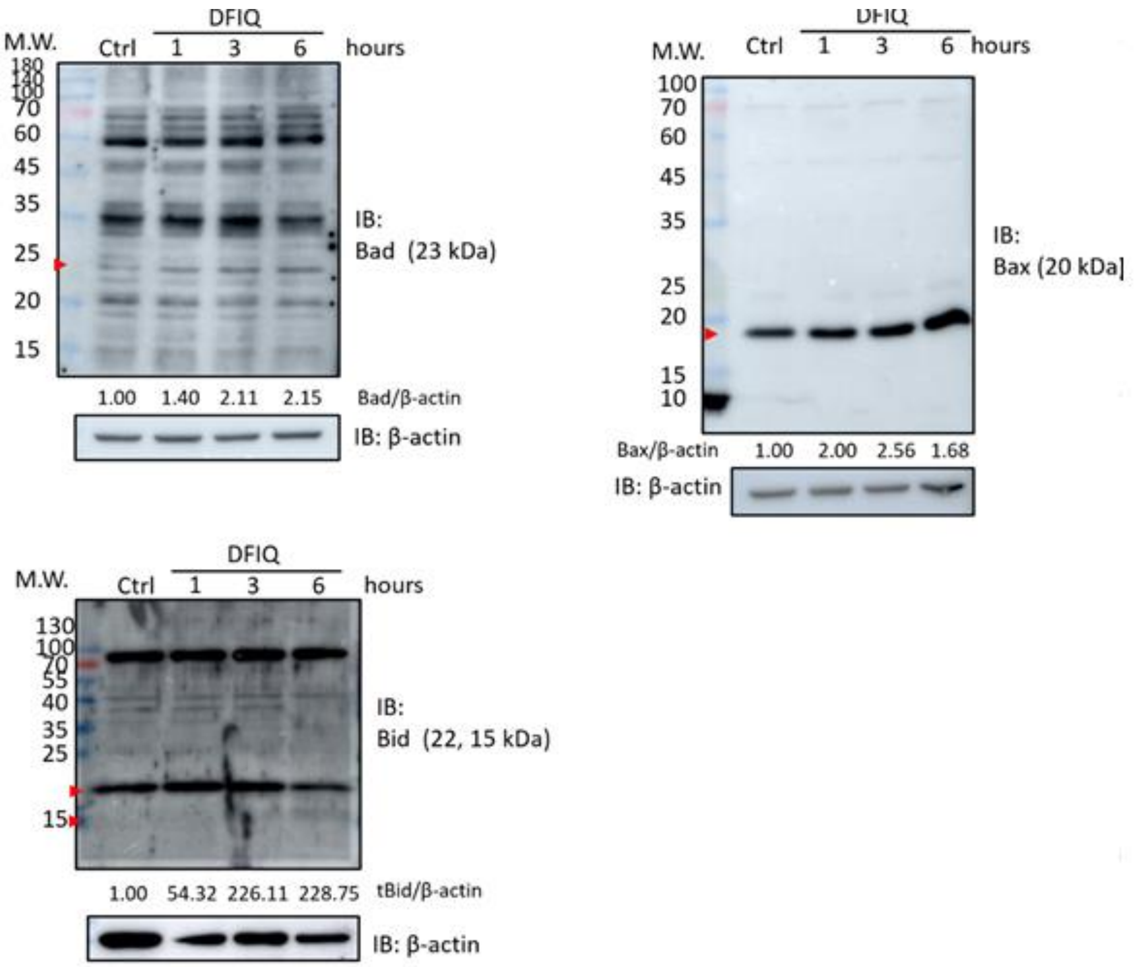

Figure S6. The uncropped blots and molecular weight markers of Figure 2D.

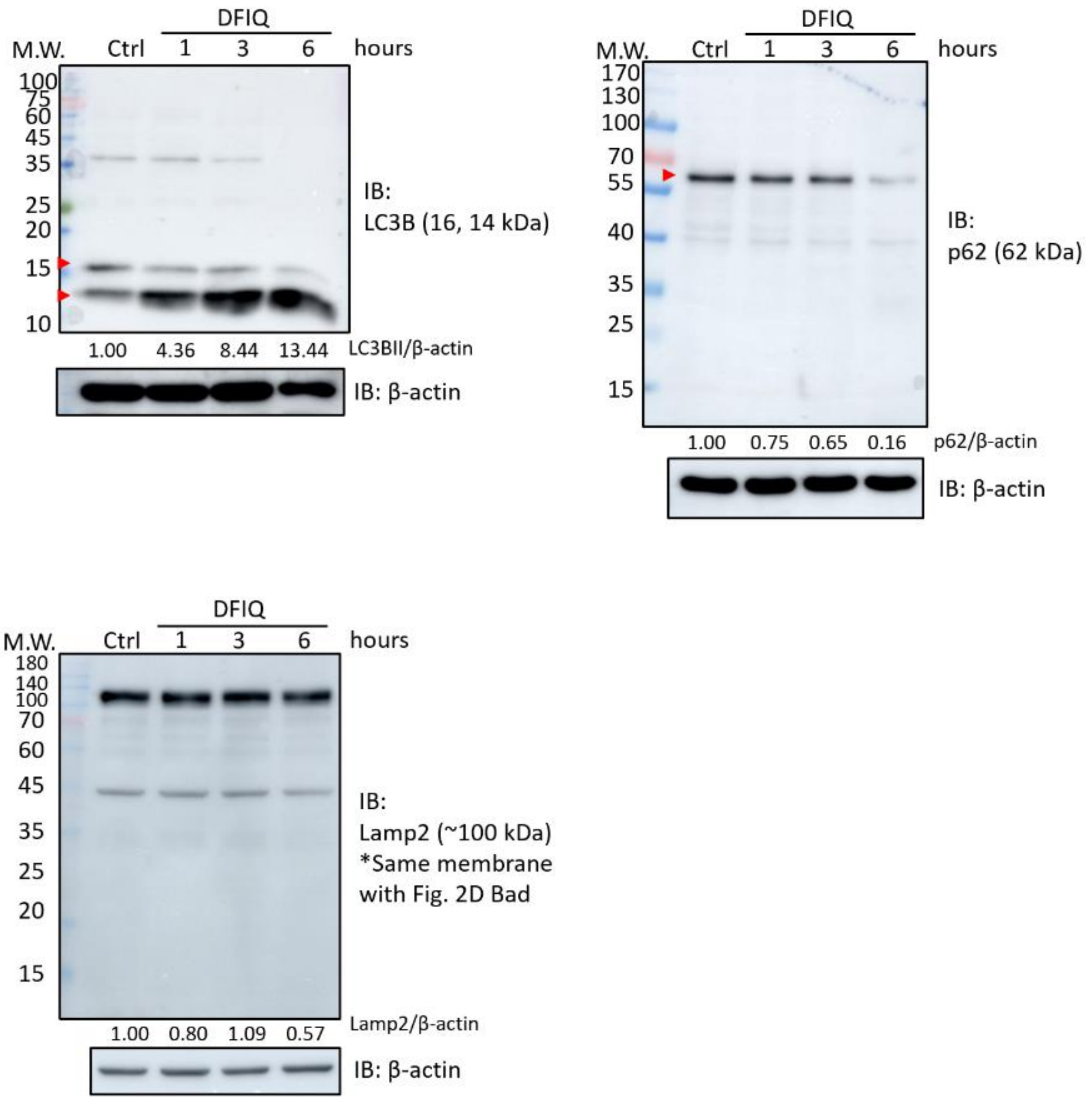

Figure S7. The uncropped blots and molecular weight markers of Figure 4A.

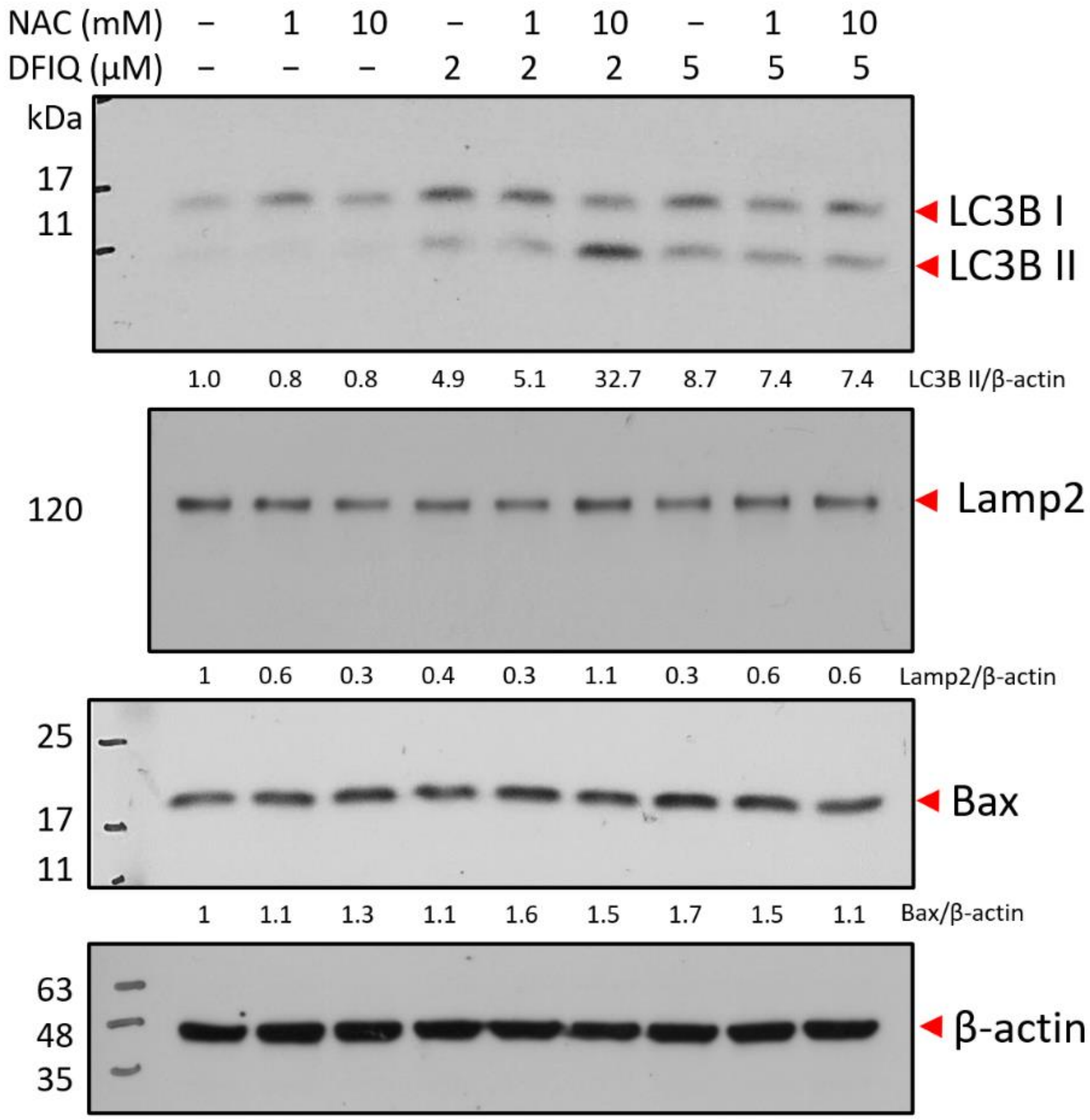

Figure S8. The uncropped blots and molecular weight markers of Figure 5A.

© 2020 by the authors. Licensee MDPI, Basel, Switzerland. This article is an open access article distributed under the terms and conditions of the Creative Commons Attribution (CC BY) license (http://creativecommons.org/licenses/by/4.0/).
